# Dynamic perfusion digital radiography for predicting pulmonary function after lung cancer resection

**DOI:** 10.1186/s12957-021-02158-w

**Published:** 2021-02-09

**Authors:** Jun Hanaoka, Makoto Yoden, Kazuki Hayashi, Takuya Shiratori, Keigo Okamoto, Ryosuke Kaku, Yo Kawaguchi, Yasuhiko Ohshio, Akinaga Sonoda

**Affiliations:** 1 Division of General Thoracic Surgery, Department of Surgery, Shiga University of Medical Science, Shiga, Japan; 2 Department of Radiology, Shiga University of Medical Science, Shiga, Japan

**Keywords:** Dynamic perfusion digital radiography, Dynamic chest radiography, Pulmonary perfusion scintigraphy, Pulmonary perfusion, Prediction of postoperative respiratory function

## Abstract

**Background:**

Accurate prediction of postoperative pulmonary function is important for ensuring the safety of patients undergoing radical resection for lung cancer. Dynamic perfusion digital radiography is an excellent and easy imaging method for detecting blood flow in the lung compared with the less-convenient conventional lung perfusion scintigraphy. As such, the present study aimed to confirm whether dynamic perfusion digital radiography can be evaluated in comparison with pulmonary perfusion scintigraphy in predicting early postoperative pulmonary function and complications.

**Methods:**

Dynamic perfusion digital radiography and spirometry were performed before and 1 and 3 months after radical resection for lung cancer. Correlation coefficients between blood flow ratios calculated using dynamic perfusion digital radiography and pulmonary perfusion scintigraphy were then confirmed in the same cases. In all patients who underwent dynamic perfusion digital radiography, the correlation predicted values calculated from the blood flow ratio, and measured values were examined. Furthermore, ppo%FEV1 or ppo%DLco values, which indicated the risk for perioperative complications, were examined.

**Results:**

A total of 52 participants who satisfied the inclusion criteria were analyzed. Blood flow ratios measured using pulmonary perfusion scintigraphy and dynamic perfusion digital radiography showed excellent correlation and acceptable predictive accuracy. Correlation coefficients between predicted FEV1 values obtained from dynamic perfusion digital radiography or pulmonary perfusion scintigraphy and actual measured values were similar. All patients who underwent dynamic perfusion digital radiography showed excellent correlation between predicted values and those measured using spirometry. A significant difference in ppo%DLco was observed for respiratory complications but not cardiovascular complications.

**Conclusions:**

Our study demonstrated that dynamic perfusion digital radiography can be a suitable alternative to pulmonary perfusion scintigraphy given its ability for predicting postoperative values and the risk for postoperative respiratory complications. Furthermore, it seemed to be an excellent modality because of its advantages, such as simplicity, low cost, and ease in obtaining in-depth respiratory functional information.

**Trial registration:**

Registered at UMIN on October 25, 2017. https://upload.umin.ac.jp/cgi-open-bin/ctr/ctr_his_list.cgi?recptno=R000033957

Registration number: UMIN000029716

**Supplementary Information:**

The online version contains supplementary material available at 10.1186/s12957-021-02158-w.

## Background

Lung cancer, one of the most commonly diagnosed cancers, remains the leading cause of cancer-related death across the world [1]. Although a number of therapeutic options for lung cancer, such as surgery, radiotherapy, and chemotherapy, are available, pulmonary lobectomy with lymph node dissection is necessary for the treatment of primary non-small-cell lung cancer. Considering that respiratory impairment after lung resection is unavoidable, preoperative assessment of cardiovascular and lung functions is important for ensuring perioperative safety and maintenance of the activities of daily living [1–3]. Especially considering the increasing number of elderly patients or those with coexisting obstructive lung disease or interstitial lung disease related to smoking, it is important to ensure safety throughout the surgical procedure and maintenance of the daily living activities [1]. Predicting postoperative lung function involves predicting the actual values [4, 5] and evaluating the risk factors for mortality and morbidity after the surgery [2, 3]. Spirometry, diffusing capacity for carbon monoxide (DLco), and maximal oxygen consumption are important for determining a patient’s preoperative respiratory function. In clinical practice, a predicted postoperative percent forced expiratory volume in 1 s (ppo%FEV1) calculated using a method based on lung segment counting and pulmonary perfusion scintigraphy (PPS) by quantifying the pulmonary blood flow distribution to improve the predictive accuracy has shown good correlation with the actual measured postoperative values [4–10]. Furthermore, ppo%FEV1 and ppo%DLco have shown a strong correlation with long-term postoperative prognosis [2] and have been used in risk assessment algorithms created using exercise stress tests as an index [3]. Although angiography, ultrasonography, quantitative computed tomography, and dynamic magnetic resonance imaging have been used to visualize or quantify the blood flow in the lungs [11–13], PPS has been considered as the most reliable and conventional method for measuring the pulmonary blood distribution. The American College of Chest Physicians guidelines recommend assessing the risk for surgery using a functional algorithm, including ppo%FEV1 and ppo%DLco values as calculated using quantitative radionuclide perfusion scanning [14]. Even the conventionally used PPS, which is highly reliable, poses various drawbacks, such as requiring significant investment in equipment, exposure dose to radioisotopes, and ingenuity in the shooting method. Dynamic chest radiography (DCR) using dynamic flat-panel detectors with a large field of view and advanced digital image processing can provide sequential chest radiographs with high temporal resolution during the respiratory cycle and allows the quantification of pulmonary blood flow distribution from the amount of pixel value changes in the lung field associated with the cardiac cycle [15]. Furthermore, computerized DCR methods, unlike other modality, can provide information regarding pulmonary ventilation and circulation by measuring the slight changes in the pixel value, without the use of contrast media or radioisotope [16]. We specifically named this method as dynamic evaluation of pulmonary circulation for “Dynamic perfusion digital radiography (DPDR)”. DPDR is considered to be a very useful examination method because it can simultaneously provide not only qualitative and quantitative information on dynamic pulmonary circulation but also information on pulmonary ventilation or movement of the diaphragm and chest wall despite its low cost, short examination time, low exposure, and simple imaging method [17]. This circulation image obtained from DCR revealed a normal pattern, which diffuses from the pulmonary arteries to the peripheral area. Nonetheless, whether this method can be used to quantify the pulmonary blood flow needs to be determined by comparing it with the conventional method for evaluating PPS.

The present study aimed to prospectively evaluate whether blood flow imaging on chest DPDR can be a viable substitute for conventional PPS in predicting postoperative pulmonary function. Accordingly, we compared pulmonary blood flow ratios (BFRs) determined through DPDR and conventional PPS and evaluated the correlation between predicted postoperative respiratory function values using DPDR and measured values.

## Methods

### Patients

**Fig. 1:**
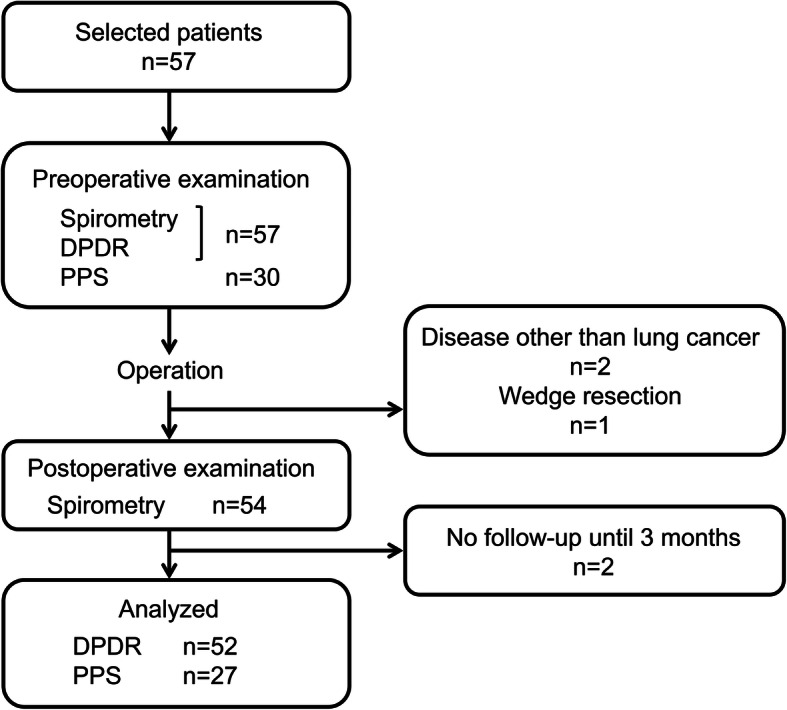
Flow diagram of the study population. Exclusion criteria: diseases other than lung cancer (*n* = 2), wedge resection of the lung (*n* = 1), lost to follow-up (*n* = 1), and refused informed consent before postoperative (*n* = 1)

This prospective study was approved by the Institutional Review Board of Shiga University of Medical Science (CRB 5180008; 10 October 2017). The study was then registered as a clinical trial (UMIN000029716). All patients provided written consent after having been informed regarding the research protocol. Patients scheduled for radical resection due to primary lung cancer from May 2018 to November 2019 were recruited. Only those who were able to follow the breathing instructions (which involved breath holding or forced breathing) in a standing or sitting position were included. A total of 57 patients underwent follow-up evaluation 3 months after surgery (February 2020). The exclusion criteria were as follows: (i) patients with a history of thoracic surgery, (ii) those younger than 20 years old, or (iii) those who may develop adverse events due to irradiation. Five patients were excluded due the following reasons: presence of disease other than lung cancer (*n* = 2), wedge resection of the lung (*n* = 1), lost to follow-up (*n* = 1), and refused informed consent before the postoperative period (*n* = 1). A total of 52 participants (40 men, 12 women; mean age 71.7 ± 7.6 years; age range 53–83 years) were ultimately analyzed (Fig. 1). Among these, 27 underwent PPS before surgery. Patient data collected included age, sex, affected side, lung lobe resected, and complications related to cardiovascular and respiratory systems.

### Methods

#### Imaging protocol for dynamic chest radiology

Posteroanterior DCR was performed using a prototype system (Konica Minolta, Inc., Tokyo, Japan) composed of an indirect-conversion flat-panel detector (PaxScan, 4343CB, Varex Imaging Corporation, Salt Lake City, UT, USA), an X-ray tube (RAD-94/B-130H, Varian Medical Systems, Inc., Palo Alto, CA, USA), and a pulsed X-ray generator (EPS45RF, EMD Technologies, Saint-Eustache, Canada). All participants were scanned in a sitting position for approximately 10 s while holding their breath. The exposure conditions were as follows: tube voltage, 100 kV; tube current, 40 mA; duration of pulsed X-ray, 5 ms; source-to-image distance, 2 m; additional filter, 0.5 mm Al + 0.1 mm Cu. Matrix size was 1024 × 1024 pixels, pixel size was 417 × 417 μm, and the whole image area was 42.7 × 42.7 cm. The pixel value range in each flat-panel detector pixel was 65,536 (16 bit). However, the pixel value was saturated at approximately 58,000, which corresponds to an entrance surface dose of approximately 1.5 μGy. A high frame rate (15 frames/s) was used for capturing perfusion-induced changes in pixel value. The pulsed X-ray protected subjects from excessive radiation exposure. Total radiation exposure was set to be <1.5 mGy, which is the International Atomic Energy Agency guidance level for both posterior anterior and lateral chest radiographies.

#### Pulmonary perfusion analysis

**Fig. 2:**
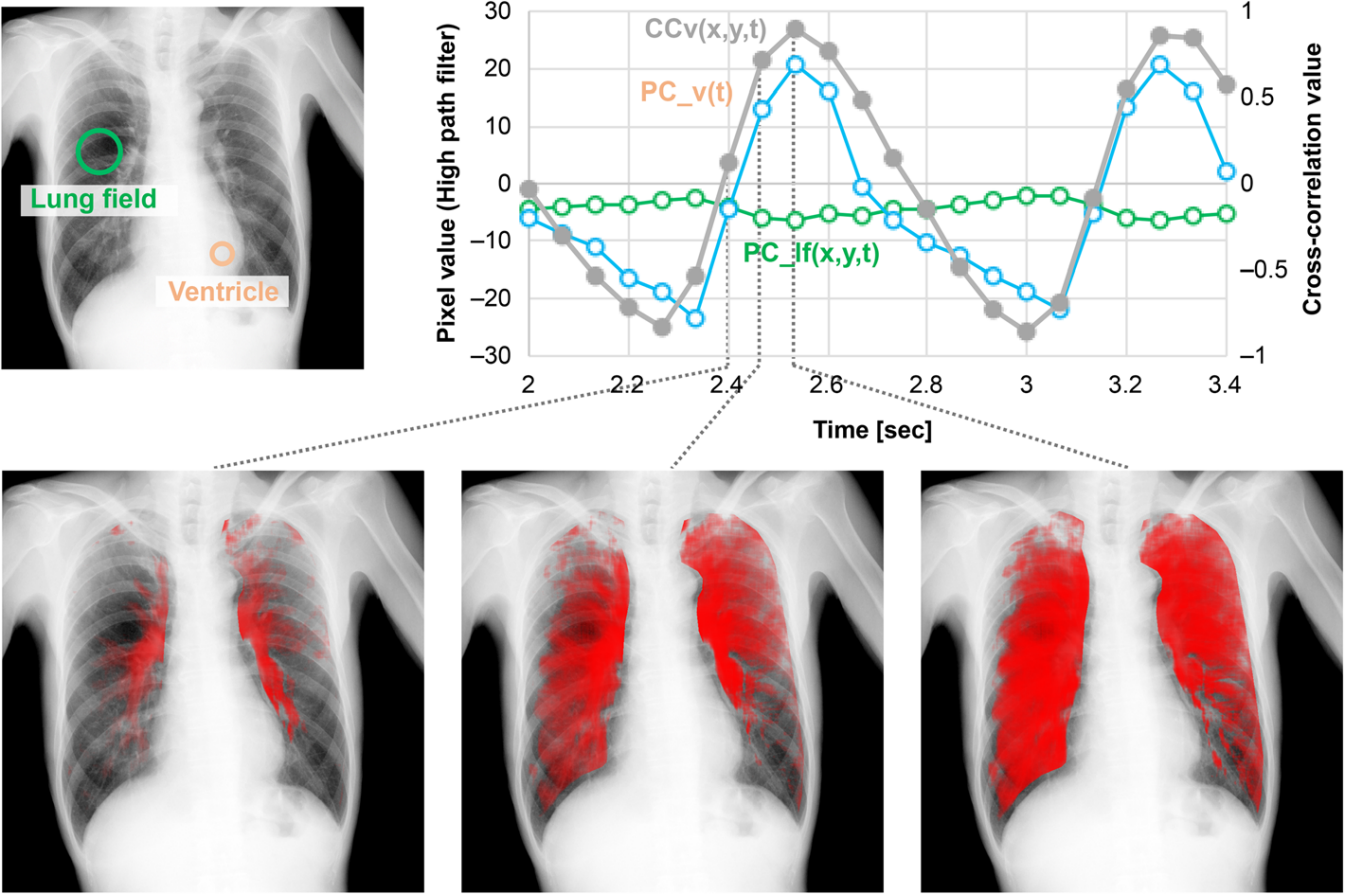
Pulmonary perfusion imaging. Pulmonary perfusion (red line) was evaluated by visualizing the degree of waveform correlation value between pixel value changes in the lung regions (green line) and periodic pixel value changes corresponding to the cardiac cycle (blue line) under cross-correlation calculation processing. Cross-correlation value changes are displayed in shades of red on each frame of the chest dynamic image

The similarity between pixel value changes in the ventricle (heartbeat waveform) and those in the lungs was determined using the following cross-correlation method. First, pixel value changes corresponding to a respiratory cycle were removed using a high-pass filter (cutoff frequency 0.85 Hz) to extract a periodic pixel value change corresponding to a cardiac cycle. Second, a 25 × 25 mm region of interest (ROI) was established on the ventricle region, after which the temporal change in pixel value was measured (PC_lv(*t*)). Last, the temporal change in pixel value was measured on each pixel at (*x*,*y*) in the lung field (PC_lf(*x*,*y*,*t*)). The cross-correlation value (CCv) between −1.0 × PC_lv and PC_lf for all pixels in the lung field was calculated frame by frame (PC_lv should be an inverse of PC_lf). The obtained CCv (from −1.0 to 1.0) at each frame was color-coded. The presence of a waveform shape similar to PC_lv indicates the presence of blood flow. A lower correlation indicates less blood flow, while a higher correlation indicates more blood flow (Fig. 2).

#### Blood flow ratio measurement

The maximum CCv (MaxCCv) of each pixel was calculated for all frames, after which the sum of MaxCCv for both left and right lung fields were calculated (SumMaxCCv_left, SumMaxCCv_right). BFR was defined as follows when the left lung was affected (Eq. 1) and when the right lung was affected (Eq. 2):

1 $$ \mathrm{BFR}=\mathrm{SumMaxCCv}\_\mathrm{left}/\left(\mathrm{SumMaxCCv}\_\mathrm{right}+\mathrm{SumMaxCCv}\_\mathrm{left}\right) $$

2 $$ \mathrm{BFR}=\mathrm{SumMaxCCv}\_\mathrm{right}/\left(\mathrm{SumMaxCCv}\_\mathrm{right}+\mathrm{SumMaxCCv}\_\mathrm{left}\right) $$

#### Pulmonary perfusion scintigraphy

A dual-head, variable-angle gamma camera (Discovery630; GE Healthcare Life Sciences, Amersham Place, Little Chalfont, Buckinghamshire HP7 9NA, England) with high-resolution low-energy collimators acquired with a 256 × 256 matrix size (zoom 1.0) was used to perform lung perfusion scintigraphy. To ensure optimal radiopharmaceutical distribution in the lungs, each patient initially received half of the 200-MBq 99mTc microalbumin/99mTc-macroaggregate solution in prone position, followed by the other half with the patient in supine position. After intravenous administration, planar scans were obtained in eight projections: anterior, posterior, left lateral, right lateral, right anterior oblique, right posterior oblique, left anterior oblique, and left posterior oblique (1 million counts each). Images were processed using an automated software for quantitative perfusion analysis which was available at a Xeleris image-processing station. This software automatically divides both lungs into three regions of interest in AP and PA projections and calculates geometric mean values from both projections in all six ROIs.

#### Pulmonary function tests

All participants underwent pulmonary function tests within 2 months prior to surgery and at 1 and 3 months after surgery using a computerized spirometer (FUDAC-77; Fukuda Denshi Co., LTD, Tokyo, Japan). Pulmonary function test parameters included FEV1, %FEV1, DLco, and %DLco values. From the perspective of the frequency of postoperative complications, we selected 1 month after the surgery as an index of surgical safety and 3 months after the surgery as reflecting a high frequency of complications [18, 19]. At 3 months after surgery, the effects of surgery in several cases seemed withdrawn with the recovery of the postoperative lung functions.

#### Predicted postoperative lung function using the segment-counting method

Predicted postoperative lung function values were calculated using a segment-counting formula developed by Ali et al. [20] and Gass GD et al. [21] (Eq. 3):

3 $$ \mathrm{ppoFEV}1=\mathrm{preFEV}1\times \frac{\mathrm{Remaining}\ \mathrm{lung}\ \mathrm{segment}\ \mathrm{numbers}}{\mathrm{Total}\ \mathrm{lung}\ \mathrm{segment}\ \mathrm{numbers}} $$

In the segment-counting method, “a” represents the total number of unobstructed segments in the resection lobe, which is assumed to be 3, 2, and 5 for the right upper, middle, and lower lobes, respectively, and 3, 2, and 4 for the left upper segments, lingular segment, and lower lobe, respectively. Overall, 19 segments were used to represent the entire lung. Furthermore, predicted postoperative lung function values were calculated using the left–right BFR from DPDR and PPS through the following formula (Eq. 4):

4 $$ \mathrm{ppoFEV}1\ \left(\mathrm{DPDR}\ \mathrm{or}\ \mathrm{PPS}\right)=\mathrm{preFEV}1\times \left(\mathrm{BFR}\ \mathrm{of}\ \mathrm{intact}\ \mathrm{side}+\frac{\mathrm{Remaining}\ \mathrm{lung}\ \mathrm{segment}\ \mathrm{number}}{\mathrm{Total}\ \mathrm{lung}\ \mathrm{segment}\ \mathrm{number}\ \mathrm{of}\ \mathrm{affected}\ \mathrm{side}}\times \mathrm{BFR}\ \mathrm{of}\ \mathrm{affected}\ \mathrm{side}\right) $$

Correlations between each predicted postoperative value and actual spirometry value were examined at 1 and 3 months after the surgery, with ppoDLco (DPDR or PPS) being calculated at the same time points.

#### Statistical analysis

All statistical analyses were performed using SPSS version 25 (Chicago, IL, USA). Data were reported as means ± standard deviations (SDs). Correlations between BFRs calculated through DPDR and PPS before surgery were evaluated using Pearson’s correlation and regression analysis. Furthermore, one version of the Bland–Altman analysis [22] was used to compare BFRs calculated through DPDR and PPS (with certain systematic and random errors). Intervals of agreement were drawn as the mean difference between predicted and measured values ± 2SD of the differences. Correlations between postoperatively measured values and those predicted using the BFR from DPDR and PPS at postoperative months 1 and 3 were examined. Furthermore, the correlation between values predicted using DCR and those measured at postoperative months 1 and 3 were analyzed. Differences between ppo%FEV1 and ppo%DLco, which indicate risk for perioperative respiratory complications, were assessed using the unpaired *t* test. All statistical analyses were two-tailed, with significance being set at 0.05.

## Results

### Clinical characteristics

A total of 52 patients were analyzed throughout the study period (Fig. 1). Of the total, 50 underwent lobectomy, and two underwent segmentectomy (upper division, S3, and S6). Their clinical characteristics are summarized in Table 1. A total of 30 patients underwent PPS before surgery, among whom 27 were suitable for final analysis. The resected lobes included the right upper (*n* = 18), right middle (*n* = 4), right lower (*n* = 8), left upper (*n* = 16), and left lower (*n* = 6) lobes. Complications requiring treatment up to 3 months after surgery occurred in 19 cases, among which 14 were respiratory system-related (bacterial pneumonia, interstitial pneumonia, pleuritis, atelectasis, hypoxemia, and prolonged air leakage) and six were cardiovascular system-related (paroxysmal atrial fibrillation). During this observation period, there was no recurrence in the eligible cases, and neither death due to other disease nor death due to cancer occurred.

**Table 1:** Patients’ clinical characteristics

Variable	No. of patients			
	With PPS	(%)	Total	(%)
Cases	27	(100.0)	52	(100.0)
Age (years), mean ± SD	73.0 ± 6.8		71.7 ± 7.6	
Gender				
Male	24	(88.9)	40	(76.9)
Female	3	(11.1)	12	(23.1)
Affected side				
Right Left	12 15	(44.4) (55.6)	30 22	(57.7) (42.3)
Resection				
RU	6	(22.2)	18	(34.6)
RM	2	(7.4)	4	(7.7)
RL LU LL	4 10 5	(14.8) (37.0) (18.5)	8 16 6	(15.4) (30.8) (11.5)
Complications Respiratory Cardiovascular	10 10 1	(37.0) (37.0) (3.7)	19 14 6	(36.5) (26.9) (11.5)

*PPS* pulmonary perfusion scintigraphy, *RU* right upper lobe, *RM* right middle lobe, *RL* right lower lobe, *LU* left upper lobe, *LL* left lower lobe

### Blood flow distribution

**Fig. 3:**
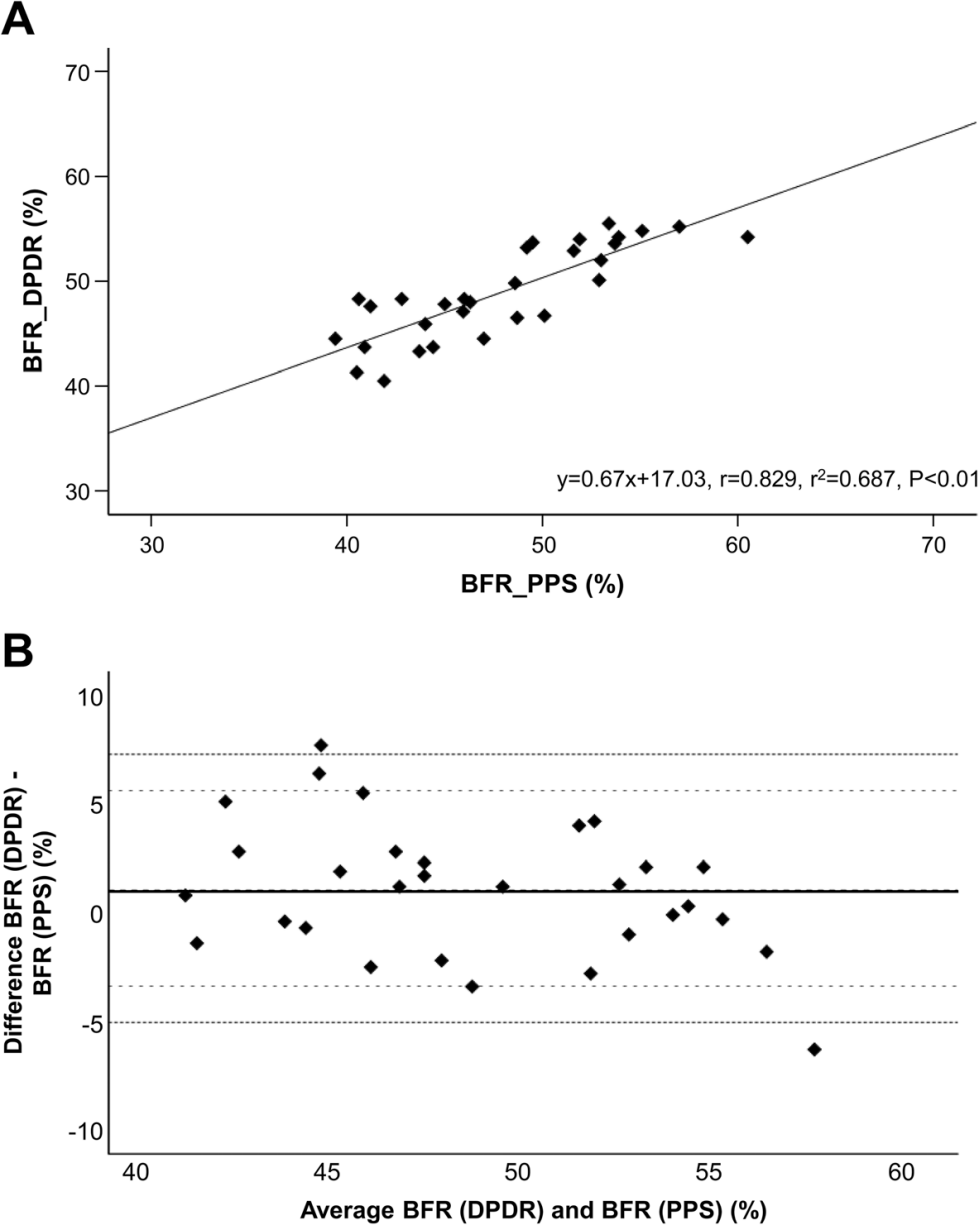
Comparison between blood flow ratio (BFR) from dynamic perfusion digital radiography and pulmonary perfusion scintigraphy. **a** Correlation between the BFR on the affected side obtained from dynamic perfusion digital radiography (DPDR) and pulmonary perfusion scintigraphy (PPS). **b** Bland–Altman analysis between affected side BFR obtained from DPDR and PPS. The black line indicates the mean, the dotted lines indicate the limit of agreement, and the broken lines indicate mean ± 2SD

The correlation between affected side BFRs obtained from DPDR and PPS was examined in 30 patients before surgery. Figure 3a shows the linear regression analysis of the affected side-to-total ratio obtained using PPS and DPDR. Accordingly, the PPS distribution ratio (*y*) and DPDR ratio (*x*) showed excellent correlation (*r* = 0.829; *P* < 0.01). Bland–Altman analysis revealed a 1.02% difference in the means of 93.3% of the values within 2 SDs of the mean and a proportional error indicating a regression line of *y* = −0.238*x* + 12.535 occurring due to variations in the smaller average side of the scatter diagram (Fig. 3b). This was attributed to substantial variation in BFRs between DPDR and PPS on the left affected side, which was caused by the effects of a beating heart during the analysis. Based on the above, the BFRs obtained from DPDR and PPS showed a high correlation and were within an acceptable error range, suggesting that DPDR was comparable to PPS in blood flow evaluation.

### Relationship between values measured using spirometry and those predicted using DPDR or PPS

**Fig. 4:**
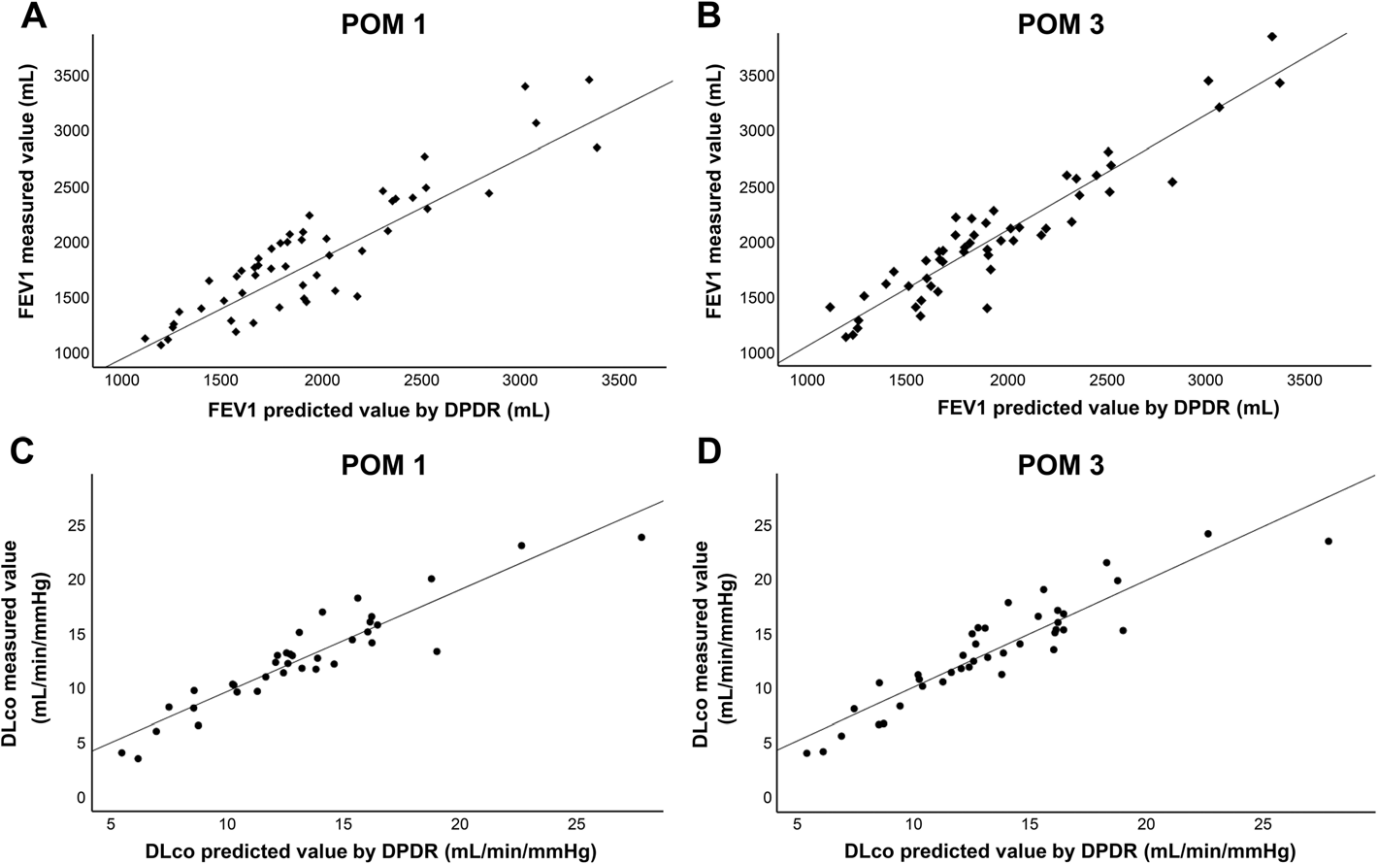
Comparison between postoperative predicted and measured values of FEV1 and DLco. Correlations between actual postoperatively measured values and those predicted using dynamic perfusion digital radiography (DPDR) for the forced expiratory volume in 1 s (FEV1) and diffusing capacity for carbon monoxide (DLco) at postoperative months 1 (**a**, **c**) and 3 (**b**, **d**)

The relationship between the actual measured values and those predicted using BFRs obtained from DPDR and PPS was examined among 27 patients who underwent simultaneous DPDR and PPS. The correlation between FEV1 and DLco values measured using spirometry, which have been frequently used to determine postoperative functional lung capacity and predicted values was examined at postoperative months 1 and 3. Accordingly, the correlation coefficients between predicted values obtained from DPDR or PPS and actual measured values at postoperative months 1 and 3 were similar for FEV1 (0.885 and 0.915 on DPDR; 0.895 and 0.915 on PPS, respectively) (Additional file 1). Table 2 presents a summary of the data obtained using DPDR and PPS. Our results also showed high correlation between DPDR and PPS for DLco, with higher correlation coefficients for FEV1 and DLco at postoperative months 3 and 1, respectively. The evaluation of perioperative respiratory status was useful in the early stage of DLco and in the stable period of FEV1. Based on these results, the correlation between values predicted using DPDR in all 52 patients and those measured using spirometry at postoperative months 1 and 3 were examined. Linear regression analysis of actual measured values at postoperative months 1 and 3 for FEV1 and DLco showed excellent correlation (*r*^2^ = 0.816, *y* = 0.945*x* + 25.836; *r*^2^ = 0.883, *y* = 1.034*x* + (−2.669); *r*^2^ = 0.862, *y* = 0.942*x* + 0.502; and *r*^2^ = 0.845, *y* = 0.989*x* + 0.453) (Fig. 4). Our results showed that values predicted using BFR calculated from DPDR was highly correlated with those predicted using PPS and those actually measured during the early postoperative period. Based on the above, the postoperative pulmonary function predicted from DPDR and PPS respectively showed a high correlation with the actual measured value, and there was no difference. Moreover, especially the correlation of the predicted value using DPDR did not decrease even when the number of samples increased, suggesting that DPDR was comparable to PPS in postoperative lung function prediction.

**Table 2:** Summary of the correlation coefficients between predicted values and actual measured values after surgery

	FEV1		DLco	
	POM 1	POM 3	POM 1	POM 3
DPDR	0.885	0.951	0.929	0.915
PPS	0.895	0.951	0.930	0.917

*DPDR* dynamic perfusion digital radiography, *PPS* pulmonary perfusion scintigraphy, *FEV1* forced expiratory volume in 1 s, *DLco* diffusing capacity for carbon monoxide, *POM* postoperative month

### Predicting postoperative complications through predictive lung function

**Fig. 5:**
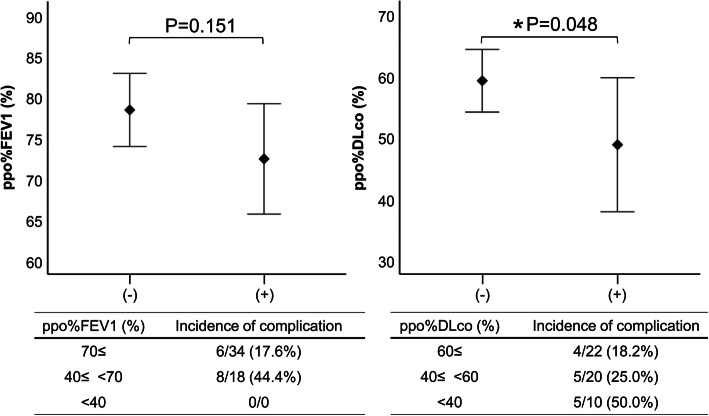
Relationship between perioperative complications and postoperative predictive value. Differences between predicted postoperative % forced expiratory volume in 1 s (ppo%FEV1) and predicted postoperative % diffusing capacity for carbon monoxide (ppo%DLco) for the prediction of perioperative respiratory complications

A total of 19 patients (36.5%) developed postoperative complications requiring treatment, among whom 14 (26.9%) were respiratory-related and 6 (11.5%) were cardiovascular-related (Table 1). The mean difference between ppo%FEV1 and ppo%DLco values obtained from DPDR, which were calculated using the prediction formula including age and height, was determined using an unpaired *t* test. Although no significant difference in cardiovascular complications was observed, a significant difference in ppo%DLco for respiratory complications was noted (Fig. 5). Complication rates for ppo%FEV1 at 40–70% and ≥70% were 44.4% and 17.6%, respectively, excluding values <40%. Gradual complication rates for ppo%DLco at <40%, 40–60%, and ≥60% were 50, 25, and 18.2%, respectively. Our results revealed that ppo%DLco reflected the risk for respiratory complications within 3 months.

## Discussion

The purpose of this study was to evaluate whether DPDR, a new technology, can be applied as a substitute for postoperative pulmonary function prediction and postoperative complication prediction using the conventional PSS.

Our results revealed a strong correlation between PPS and DPDR in the regression analysis of affected side BFR among patients with lung cancer before surgery (Fig. 3a). Furthermore, Bland–Altman analysis revealed that the limit of agreement on optimistic interpretation was between −3.386 and 5.819, indicating an acceptable degree of agreement (Fig. 3b). Moreover, the present study found that values predicted using BFR from DPDR strongly correlated with not only those predicted from PPS but also those actually measured among patients who underwent DPDR during the early postoperative period (Table 2; Fig. 4, Additional file 1). Previous studies had reported that the correlation coefficient for PPS was approximately 0.8–0.9 [5, 8–10, 23, 24], which is comparable to the results presented herein. Furthermore, our results revealed that ppo%DLco reflected the risk of respiratory complications within 3 months (Fig. 5). Although some reports show that FEV1 can be a good predictor of postoperative respiratory function, the present study only showed a trend given our exclusion of patients with a ppo%FEV1 of <40% [2, 3, 14]. The current study showed that pulmonary blood flow measurements obtained using DPDR strongly correlated with the results of blood flow scintigraphy, suggesting that DPDR can be a possible substitute for PPS in blood flow distribution analysis.

PPS has been an established method for assessing pulmonary blood flow; however, it may impose a burden on medical institutions and patients (e.g., facility investment, nuclide preparation, exposure dose, and shooting time). DCR provides visual information on respiratory kinetics and functional imaging of the pulmonary circulation and ventilation [15]. Furthermore, DPDR can be potentially advantageous considering its low dose, small space requirement, and cost effectiveness. Provided that no significant differences in the obtained results are noted, DPDR may be considered to be more convenient than PPS in a clinical setting (see Table 3 for a comparison between DPDR and PPS). The evaluation and imaging methods (procedure) have not yet been established because of a possibility that the physiological functions that can be evaluated will eventually expand.

**Table 3:** Comparison between dynamic perfusion digital radiography and perfusion scintigraphy

	Dynamic perfusion digital radiography	Perfusion scintigraphy
Cost	Low equipment cost + Work station	High equipment cost
Supplies	Unnecessary	Radionuclide:^99m^Tc-MAA,^99m^Tc-GAS
Shooting time	7 s~	20 min~
Radiation dose	0.09 mSv	0.8 mSv
Procedure	Not established	Established
Information of result	Same as X-ray image	One-sided lung and lobe
	Functional information can be obtained	
Evaluation	Not established	Established

Until date, DCR has been reported for image evaluation of chronic thromboembolic pulmonary hypertension, for the prediction of tumor from visceral pleura origin, correlation between changes in lung field area and lung function tests, and the evaluation of intrathoracic tracheal narrowing in patients with COPD [25–28]. DCR can provide information not only in the supine position but also in a more physiological respiratory state of breathing, depending on the patient’s position (standing or sitting). Ventilation imaging can observe not only atelectasis but also the ventilation distribution in the lung field, along with the observation of uneven distribution reflecting the air trapping associated with diffuse peripheral airway lesions, such as asthma. In addition, it is possible to observe not only the lung field but also the movement of the skeleton and diaphragm; therefore, it is possible to evaluate the differences in the expansion of the lung parenchyma in the upper and lower lung fields, such as IPF. Moreover, DPDR allows blood stream kinetics assessment, such as blood flow change for each heartbeat, and ventilation/perfusion ratio mismatch determined from ventilation and perfusion imaging at the same time axis. We believe that a portable DPDR system for emergency medicine within disaster areas will be made available in the diagnosis of pulmonary thromboembolism in the near future. As a result, a lot of information from the respiratory and circulatory kinetics in normal imaging and ventilation/blood flow imaging by image processing could be easily obtained, and it may be widely used for purposes from disease diagnosis to functional evaluation in the future. Thus, the study seems to provide considerable incentives for exploring the respiratory and cardiovascular physiologies [16].

The current study has some limitations. First, this was a single-center study that included a small sample size. Moreover, this study was limited to surgery cases and excluded those with poor lung function from surgery, which likely resulted in bias. Furthermore, preoperative PPS was not performed in all cases to minimize exposure. Instead, it was only performed in cases with ventilatory and/or diffusion impairment. Second, DPDR does not directly measure actual lung perfusion but provides relative functional information related to lung circulation. Thus, some differences between DPDR and PPS findings are not surprising as both have completely different imaging targets, imaging mechanisms, and imaging postures. For instance, DPDR relies on pulmonary arterial blood flow to capture pulsations; whereas, PPS captures the accumulation of ^99m^Tc-MAA microemboli in peripheral capillaries. Thus, examining cases with different degrees of pulmonary function will be essential in the future to reveal more accurate correlations.

## Conclusions

Our study demonstrates that DPDR, which is simple, inexpensive, and makes obtaining respiratory functional information easy, can be a suitable substitute for PPS to predict postoperative values and the risk for postoperative respiratory complications. Nonetheless, further multicenter studies with larger sample sizes are needed to confirm our findings. This study provides novel insights using a new imaging system and demonstrates the possibility for functional analysis using DPDR.

## Supplementary Information

**Additional file 1.** Comparison between predicted and measured values on forced expiratory volume in 1 s (FEV1). Correlations between FEV1 values predicted from dynamic perfusion digital radiography (DPDR) and pulmonary perfusion scintigraphy (PPS) and those measured using spirometry at postoperative months 1 (a, c) and 3 (b, d)

## Data Availability

The datasets used and/or analyzed during the current study are available from the corresponding authors on reasonable request.

## Abbreviations

DCR: Dynamic chest radiography
DPDR: Dynamic perfusion digital radiography
PPS: Pulmonary perfusion scintigraphy
BFR: Blood flow ratio
FEV1: Forced expiratory volume in 1 s
DLco: Diffusing capacity for carbon monoxide
