# Supplementary figures and images for: Dynamic perfusion digital radiography for predicting pulmonary function after lung cancer resection
Source: World J Surg Oncol. 2021 Feb 9;19:43. doi: 10.1186/s12957-021-02158-w (PMC7874664; doi:10.1186/s12957-021-02158-w)

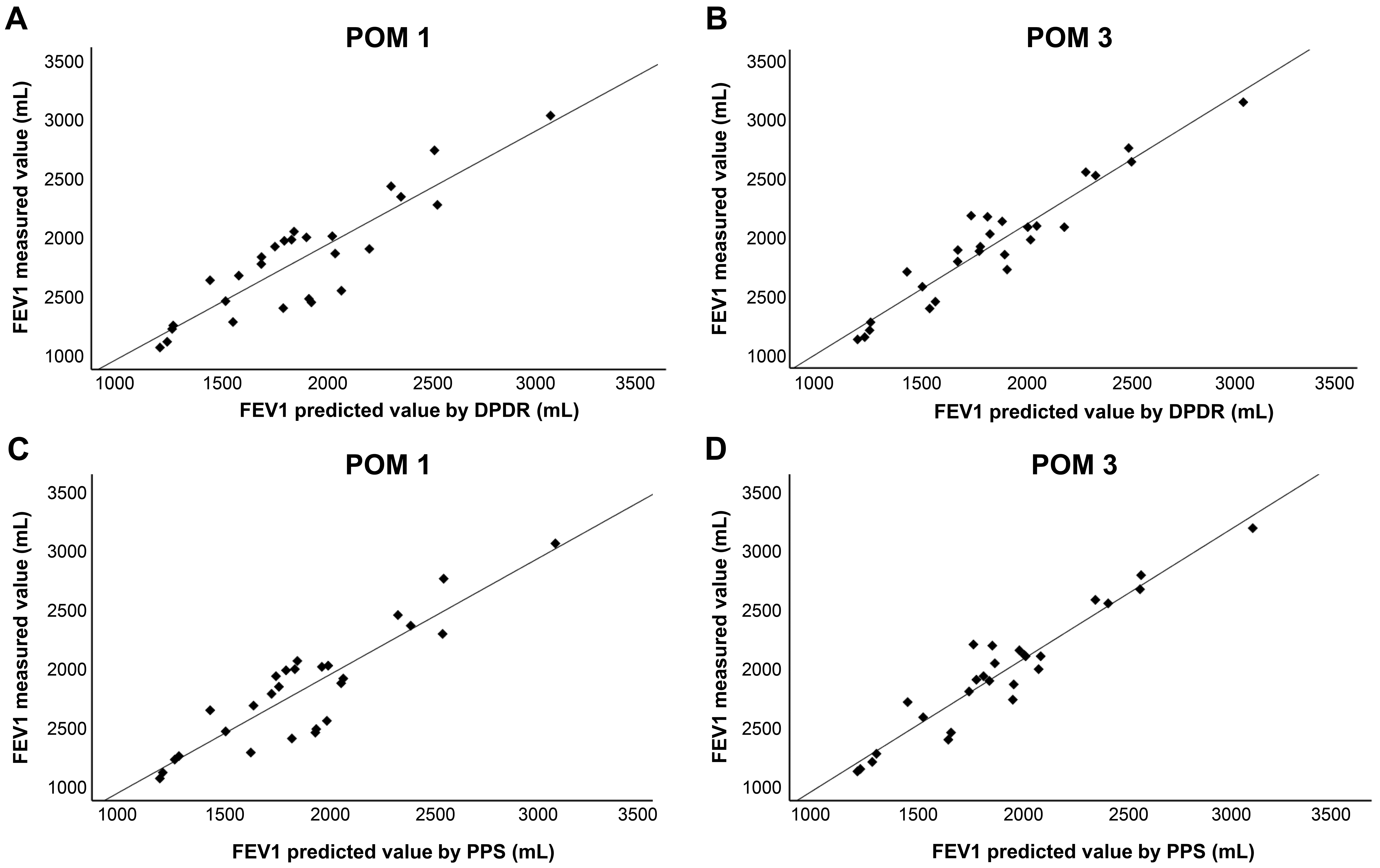

Supplement: Supplementary file 1 — Additional file 1. Comparison between predicted and measured values on forced expiratory volume in 1 s (FEV1). Correlations between FEV1 values predicted from dynamic perfusion digital radiography (DPDR) and pulmonary perfusion scintigraphy (PPS) and those measured using spirometry at postoperative months 1 (a, c) and 3 (b, d) [file 12957_2021_2158_MOESM1_ESM.tif]
